# Vimentin 3 Allows Differentiation between Normozoospermia and Oligoasthenoteratozoospermia

**DOI:** 10.1155/2019/9803498

**Published:** 2019-12-10

**Authors:** Timo Funke, Melanie von Brandenstein, Pia Paffenholz, Barbara Köditz, Tim Nestler, Jan Herden, Manuel Montesinos-Rongen, Evgenia Isachenko, Gohar Rahimi, Jochen W. U. Fries, Axel Heidenreich, Johannes Salem

**Affiliations:** ^1^Department of Urology, University Hospital of Cologne, Germany; ^2^Department of Neuropathology, University Hospital of Cologne, Germany; ^3^Department of Gynecology and Obstetrics, University Hospital of Cologne, Germany; ^4^Department of Pathology, University Hospital of Cologne, Germany

## Abstract

Vimentin is a structural protein predominantly located in the head of sperms. The function and localization of the previously identified truncated version, Vimentin 3 (Vim3), are still unknown. To investigate whether the expression of Vim3 can be used as a reliable marker for the differentiation of sperm quality, we analyzed ejaculates from patients with oligoasthenoteratozoospermia (OAT) syndrome and normozoospermia. We identified sperms with head, neck, and tail changes, which were less positive for Vim3 in OAT syndrome compared to normozoospermia. The expression of Vim3 was significantly downregulated in patients with OAT syndrome compared to sperms from patients with normozoospermia (^∗∗^*p* < 0.01). The ELISA analysis showed similar results as ejaculates from normozoospermic patients showed a significantly higher Vim3 concentration than patients with OAT syndrome (^∗∗∗^*p* < 0.001). This study demonstrates that Vim3 is more highly expressed in ejaculates from patients with normozoospermia compared to ejaculates from patients with OAT syndrome. Therefore, we postulate that Vim3 can be used to determine ejaculate quality. Furthermore, we identified the marker, Vim3, to differentiate between mature sperms with no morphological changes and sperms with head, neck, and tail changes. A lateral flow assay that allows quick analysis is currently under development.

## 1. Introduction

Around 30 million men worldwide are infertile with the highest rates in Africa and Eastern Europe [1]. Several different causes of infertility in men exist [2]. Possible causes include gonadal disorders (30-40%), disorders affecting sperm transport (10-20%), and hypothalamic or pituitary disorders (1-2%) [3]. However, most of the causes of infertility are unknown (40-50%).

Sperm abnormalities can be caused by different factors, such as inflammation of the testis, varicoceles, abnormally developed testis, genetic disorders, or hormone problems [3].

Vimentin is a structural protein and is predominantly expressed in the head domain of sperms [4]. Prior analyses showed that an asymmetric distribution of Vimentin in sperm cells is correlated with different structural defects in spermatozoa [5].

As previously published, Endothelin-1 (ET-1) is responsible for the expression of a truncated variant of Vimentin [6], called Vimentin 3 (Vim3). This truncation process is induced by miRNA 498 binding to its complementary sequence on the DNA, which results in a transcriptional stop. This shorter mRNA strand is translated into Vim3, a biologically functional protein [7].

Consequently, the normal full-length Vimentin protein, which can be found in the head and tail domains of sperms, is no longer synthesized. It was furthermore shown that Vim3 is upregulated in benign kidney tumors [6], which are associated with the presence of high numbers of nonfunctional mitochondria. Therefore, the question has been raised whether other cells in the human body that also give rise to nonfunctional mitochondria, likewise showing Vim3 production. In sperms, mitochondria are predominantly present in the neck domain. Nekrasova et al. found that Vimentin represents an important anchor for mitochondria [8]. Maggi et al. demonstrated that normozoospermic ejaculates show the highest ET-1 expression [9]. Consequently using this method Vim3 not only can be used to identify normozoospermia but can also help to reveal if patients suffer from oligoasthenoteratozoospermia [9].

## 2. Material and Methods

### 2.1. Ejaculates

The patients' ejaculate (*n* = 27) samples were analyzed and categorized according to the nomenclature of the WHO (World Health Organization) from 2010. The study complies with the Declaration of Helsinki, and local ethics committee approval was obtained (BioMASOTA, University Hospital of Cologne, file references 12–163). All samples were taken from the present biobank (Table 1). Samples were stored at -20°C.

### 2.2. Immunofluorescence

For each ejaculate sample, 100 *μ*l was centrifuged at 2500 × *g*. The pellet was washed twice with 500 *μ*l 1x PBS (PAN-Biotech GmbH, Aidenbach, Germany) and centrifuged again at 2500 × *g*. After washing, the pellet was diluted in 25 *μ*l 1x PBS and 10 *μ*l were spread on superfrost slides (Thermo Scientific, Menzel-Gläser, Braunschweig, Germany). The slides were air-dried and washed under running tap water. No further fixation methods were used. The Vim3 (Davids Biotechnologie GmbH, Regensburg, Germany) or V9 antibody (Santa Cruz Biotechnology Inc., Dallas, USA), which detected the full-length version of Vimentin, was diluted by a factor of 1 : 1000 in 1x PBS and incubated for 1 hour. After incubation, slides were washed three times in 1x PBS. Subsequently, the second antibody (FITC anti-rabbit or Alexa 594 anti-rabbit) (Santa Cruz Biotechnology Inc., Dallas, USA) was added, incubated for 30 min, and again washed twice with 1x PBS. Finally, the slides were covered with DAPI mounting medium (Abcam, Cambridge, UK) and analyzed using the fluorescence microscope Olympus DP7. For analysis purposes, we used the DISCUS software.

### 2.3. Enzyme-Linked Immunosorbent Assay (ELISA)

ELISA was performed to determine the Vim3 or ET-1 concentration in sperm cells. Therefore, the patient samples were washed 3x times with 1x PBS. For this purpose, 96 noncoated well plates (Brand GmbH + Co KG, Wertheim, Germany) were utilized and, afterwards, 50 *μ*l of the patient samples was incubated for one hour at room temperature. The washing procedure was carried out three times using 1x PBS. Subsequently, the specific Vim3 antibody (1 : 1000) (Davids Biotechnologie GmbH, Regensburg, Germany) was incubated for one hour at room temperature. After washing was performed twice, the second antibody (1 : 5000) (Biomol GmbH, Hamburg, Germany) was also incubated for one hour at room temperature. Subsequent to performing the washing step twice again, 50 *μ*l of the TMB ELISA solution was added and the recreation phase was stopped using the stop solution (Bethyl Laboratories, Inc., Montgomery, USA). The ELISA results were obtained at 450 nm. For the quantification of the ELISA, a standard curve was produced using purified Vim3 peptide (Davids Biotechnologie GmbH, Regensburg, Germany). For the determination of the ET-1 expression, an ET-1 ELISA kit (Wuhan Fine Biotech Co. Ltd., Wuhan, China) was used according to the manufacturer's protocol.

### 2.4. Statistics

In order to differentiate between normozoospermia and oligoasthenoteratozoospermia, ejaculates were collected and stained with Vim3. The signal intensity produced by the normozoospermic samples was defined as standard, and for each sample, 100 sperms were counted. For statistical analysis purposes, we used the Prism 5 (GraphPad Software, San Diego, USA) software. A *t*-test was performed, and significant differences were calculated (^∗^*p* < 0.05, ^∗∗^*p* < 0.01, and ^∗∗∗^*p* < 0.001). All experiments were performed as triplicate.

## 3. Results

**Figure 1:**
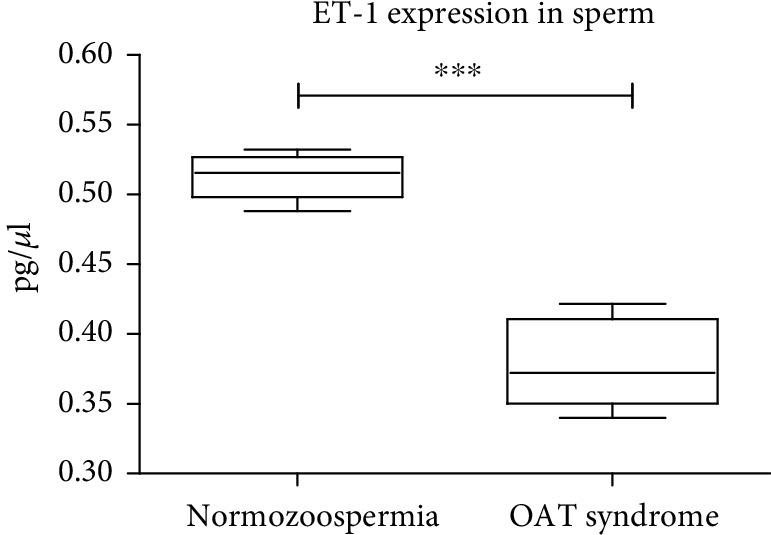
ET-1 ELISA from washed sperms showing a significant downregulation of ET-1 expression in OAT syndrome compared to normozoospermia (^∗∗∗^*p* < 0.001).

We first analyzed the expression of ET-1 in sperm cells comparing patients with normozoospermia and OAT syndrome. We found a significantly higher expression of ET-1 in normozoospermia compared to the OAT syndrome (^∗∗∗^*p* < 0.001; Figure 1).

**Figure 2:**
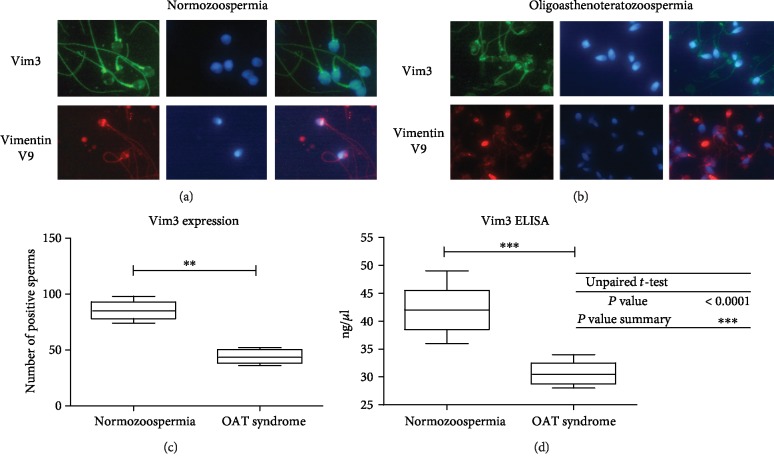
Vimentin staining of normozoospermia and OAT syndrome. (a) Immunofluorescence showing the expression of Vim3 (green) in normozoospermia (blue) indicates the sperm head (DAPI). We showed that the localization of the Vim3 is predominant in the neck and tail regions. The full-length Vimentin can be found in the lower row (red), which shows a dominant location in the head, especially in the equatorial region. (b). Immunofluorescence analysis of spermatozoa from a patient with OAT syndrome. Vim3 is predominantly detectable in the head and tail regions, whereas the distribution of the full-length variant is found in the head and therefore similar to the distribution in ejaculates from normozoospermic patients. (Magnification ×100). The most part clearly structured distribution within the equatorial region of the head. (c). Calculation of Vim3-positive sperms as shown in a boxplot. Fluorescence intensity showed a significant downregulation in Vim3 expression in OAT syndrome compared to normozoospermic patients (^∗∗^*p* < 0.01); the intensity associated with normozoospermia was defined as “standard” intensity. For each group, seven different samples were calculated, and 100 sperms were counted per group. (d). ELISA analysis of Vim3 concentration in sperm cell showed a significant downregulation of Vim3 expression in OAT syndrome compared with normozoospermic patients (^∗∗∗^*p* < 0.001).

As a next step, we analyzed the localization and expression of Vim3, which is known to be upregulated by ET-1 [10]. In patients with normozoospermia, the Vim3 distribution was predominantly present in the neck and tail regions of the sperm cells. The distribution of full-length Vimentin, on the other hand, is predominantly present in the head region of the sperm cells, especially in the equatorial segment (Figure 2(a)). However, in the OAT syndrome, we were able to detect a different distribution of the Vim3 variant in the analyzed sperm cells. In OAT syndrome, Vim3 is predominantly expressed in the head domain and to a lesser extent in the neck and tail domains (Figure 2(b)).

In order to calculate potential differences of the Vim3 expression between patients with normozoospermia and OAT syndrome, we performed immunofluorescence analyses. As depicted in Figure 2(c), we found a significant downregulation of the Vim3 expression in sperm cells from OAT syndrome patients compared to those from patients with normozoospermia (*p* < 0.001).

Figure 2(d) shows the levels of Vim3 expression in spermatozoa of patients with normozoospermia and oligoasthenoteratozoospermia. It can be seen that the Vim3 concentration in spermatozoa from patients with OAT syndrome is significantly downregulated compared to those from patients with normozoospermia (^∗∗∗^*p* < 0.001).

## 4. Discussion

The function and localization of the truncated version of Vimentin, called Vim3, in spermatozoa is still unknown. To investigate the potential role of Vim3 as a biomarker for sperm quality, we performed our study as an analysis of ejaculates from patients with OAT syndrome and normozoospermia.

We found that Vim3 accurately and reliably helps to differentiate between mature sperm cells and sperm cells with an abnormal shape. Furthermore, Vim3 protein shows reliable results for the differentiation between normozoospermia and oligoasthenoteratozoospermia, even in frozen samples. This is an important procedural improvement, because the patient no longer has to provide the semen sample within a restricted time period, as Vim3 can even be used in dated ejaculate samples with dead spermatozoa in it.

Our group is developing a lateral flow assay that allows the quick analysis of ejaculates, prior to performing time-consuming semen evaluation. In addition, the time between the first suspicion of infertility and its diagnosis can be unbearably long and can therefore put a lot of strain on a relationship. Using this much quicker method of sperm analysis shortens the period of uncertainty and helps to reduce psychological pressure during the process of accurately identifying fertile sperm cells. However, until the lateral flow test is integrated into clinical practice, a classical microscopic ejaculate analysis should always be the first choice in order to compare these results further.

Furthermore, the exact function of Vim3 in sperms and the expression of this variant are currently unknown and need to be further evaluated. According to unpublished data, a correlation between increased Vim3 levels and increased mobility of cells was analyzed. Also, sperms from a performed swim up test show high Vim3 positivity (data not shown).

Limitations of this study are its small number of patients as well as its single-center design. We therefore intend to enlarge our cohort of patients leading to a larger number of samples.

## 5. Conclusion

The analysis of sperm cells with the newly developed Vim3 antibody accurately and reliably helps to differentiate mature sperm cells from sperm cells with an abnormal shape. To the best of our knowledge, Vim3 is the first marker that gives reliable insight into the formation of sperms, since its expression is significantly decreased in sperm cells with head, neck, and tail changes. Furthermore, routine staining techniques can be more time-consuming than the immunofluorescence staining.

## Figures and Tables

**Table 1:** Ejaculate samples.

Age	Normozoospermia	OAT syndrome	Disease	Sperms (Mio/ml)	Volume	Mobility *a* + *b*	Mobility *c*	Mobility *d*	Normal shape
32	X		Wish to have children	290	3.5	49	8	43	5
34	X		Wish to have children	183	3	66	0	34	5
23	X		Testicular cancer	98	2	61	8	33	5
26	X		Wish to have children	427	3.5	59	11	30	5
28	X		Varicocele	137	2.5	72	4	24	4
39	X		Wish to have children	209	2.2	72	5	23	4
44	X		Wish to have children	351	3	59	5	36	5
43	X		Wish to have children	207	1.5	82	10	8	5
38	X		Wish to have children	188	2.5	86	5	9	5
43	X		Wish to have children	492	2	51	4	45	4
42	X		Wish to have children	385	3.5	74	2	24	4
42	X		Varicocele	216	2	62	2	36	5
36	X		Varicocele	240	2	56	4	40	5
32	X		Wish to have children	243	2.8	44	0	56	5
36	X		Wish to have children	189	2.2	47	0	53	8
34		X	Testicular cancer	11	3.5	18	0	82	1
45		X	Wish to have children	14	2.5	26	8	66	1
41		X	Multiple sclerosis	11	4	23	0	77	2
28		X	Testicular cancer	12	2.5	25	8	59	3
44		X	Nicotine abuse	5	5	17	6	77	1
33		X	Type 1 diabetes	15	3	26	9	65	0
26		X	Wish to have children	10	2.2	31	1	68	4
22		X	Varicocele	9	4	43	3	54	1
32		X	Type 1 diabetes	6	5	17	6	77	1
26		X	Testicular cancer	1	7	25	5	70	2
40		X	Testicular cancer	9	3	17	8	75	1
23		X	Testicular cancer	2	3.4	24	2	74	2

## Data Availability

The data (figures and patient data) used to support the findings of this study are included within the article.
